# Factors associated with the burden of family caregivers of patients with mental disorders: a cross-sectional study

**DOI:** 10.1186/s12888-017-1501-1

**Published:** 2017-10-25

**Authors:** Ana Lúcia Rezende Souza, Rafael Alves Guimarães, Daisy de Araújo Vilela, Renata Machado de Assis, Lizete Malagoni de Almeida Cavalcante Oliveira, Mariana Rezende Souza, Douglas José Nogueira, Maria Alves Barbosa

**Affiliations:** 1 Physiotherapy Course, Federal University of Goiás, Jataí, Goiás Brazil; 2 Faculty of Medicine, Federal University of Goiás, Goiânia, Goiás Brazil; 3 Institute of Tropical Pathology and Public Health, Federal University of Goiás, Goiânia, Goiás Brazil; 4 Physical Education Course, Federal University of Goiás, Jataí, Goiás Brazil; 5 Faculty of Nursing, Federal University of Goiás, Goiânia, Goiás Brazil; 6 Post Graduated Program in Social and Preventive Dentistry of the Faculty of Dentistry, Paulista State University, Araçatuba, São Paulo, Brazil

**Keywords:** Caregiver, Burden, Mental disorder, Mental disease

## Abstract

**Background:**

Caregivers are responsible for the home care of family members with mental-health disorders often experience changes in their life that can generate stress and burden. The aim of this study was to identify factors associated with the burden of caregivers of family members with mental disorders.

**Methods:**

This cross-sectional study was conducted with a non-probability sample of family caregivers, whose patients attended a community services program, the Psychosocial Care Centers, in three cities in the southwest region of Goiás State, Central Brazil. Data collection took place from June 2014 to June 2015. The participants were 281 caregivers who completed a sociodemographic questionnaire and the Zarit Burden Interview (ZBI). Bivariate analyses (t test, analysis of variance, and Pearson correlation) were performed, and variables with values of *p* < 0.10 and gender were included in a multiple-linear regression model. Values of *p* < 0.05 were considered significant.

**Results:**

The caregivers were mostly female and parents of the patients, were married, with low education, and of low income. The mean ZBI score was 27.66. The factors independently associated with caregivers’ burden were depression, being over 60 years of age, receiving no help with caregiving, recent patient crisis, contact days, and having other family members needing care.

**Conclusions:**

This study identified factors that deserve the attention of community services and can guide programs, such as family psycho-education groups, which may help to minimize or prevent the effects of burden on family caregivers responsible for patients’ home care.

## Background

Community services for the care of patients with mental disorders (Brazilian Psychosocial Care Centers - *Centros de Atenção Psicossocial* [CAPS]) were systematically introduced into the Brazilian public health-care system in 2002. They replaced the asylum model, providing a more open process that allows users to participate in social and family life. This implies that family members should participate in treatment strategies by providing home care in order to rehabilitate patients with mental disorders [1]. In this context, in general, family caregivers assume the responsibility for the physical, emotional, medical, and usually financial care of the sick relative. As a result, family caregivers of patients with mental disorders, without proper preparation, knowledge, or support of health professionals, often experience changes in their life. While the positive and negative effects of caregiving are not always visible, the care tends to produce high levels of caregiver burden [2, 3].

Thus, caregivers who provide care for family members with psychiatric illness are at potential risk for burden and consequent decrease in health status [4]. Some studies show multiple consequences of caregiver burden, such as mental-health problems (e.g., depression, anxiety, stress, and burnout syndrome), physical health deterioration (e.g., diabetes), and other negative effects (e.g., family dysfunction, social isolation, excessive use of health services, and financial problems) [5, 6]. Some evidence indicate significantly higher scores of overload in caregivers of psychiatric patients when compared to other conditions, such as other chronic diseases [5].

A number of determinants are associated with caregiver burden, which can be: (i) disease-related factors; (ii) clinical and socio-demographic factors, and (iii) social psychological factors [7]. Variables relating to patient illness include the duration of mental disorder [5, 8, 9], number of psychiatric hospitalizations [7], degree of functional impairment and neuropsychiatric symptoms [10], and crisis situations or problem behaviors [11]. Clinical and socio-demographic factors include variables related to the patient and caregiver. Some investigations show young age, male gender, and presence of other comorbidities (physical and/or psychiatric) of the patient are factors that increase caregiver burden [12, 13]. Caregiver-related variables strongly associated include older age [5, 14], female gender [7, 15], high household income [12, 15, 16], level of education [17, 18], degree of kinship with the patient [15], presence of physical (e.g., arterial hypertension [HTN], and diabetes mellitus) and/or mental comorbidities (e.g., depression and anxiety) [6, 10], as well as low quality of life [10]. Social psychological factors include low social support and family dysfunctionality [7, 19]. Furthermore, other variables positively related to the increase of burden are residence and days of contact with the patient [12, 13].

In Brazil, it is necessary to deepen the knowledge of burden in this population of caregivers. Despite increasing research in recent years [20–22], there are still few analytical studies on the predictors of burden in family caregivers of patients with mental disorders. Particularly, there is a knowledge gap in relation to the population of the Central-West Region. Data on this topic could contribute to changes in the strategies for supporting families, and thereby promote the improvement of mental health. Therefore, the present study aimed to identify factors associated with the burden of caregivers of family members with mental disorders.

## Methods

### Design and study sites

This was a cross-sectional study conducted with a non-probabilistic sample of family caregivers of patients with mental disorders enrolled in community services, the CAPS, in Goiás State in the Central-West Region of Brazil. Participants were recruited from three cities of Goiás: Jataí (88,000 inhabitants), Mineiros (53,000 inhabitants), and Rio Verde (176,000 inhabitants).

### Study participants and sample size

The participating informal caregivers, who were patients’ family members, provided care for them without any employment agreement or compensation. These caregivers may be primary or secondary caregivers and live with or separately from the person receiving care [23]. Inclusion criteria for caregivers were: (i) age 18 or over; (ii) providing care for a patient active on a health-care registry with a clinical diagnosis of mental disorder, according to the International Classification of Diseases (ICD-10) [24], with codes F-00 to F-99; (iii) having a kinship or affective relationship with the patient; and (iv) being the primary caregiver identified by health professionals. Exclusion criteria were as follows: (i) not able to understand the questions; (ii) under age 18; (iii) less than two months as caregiver; (iv) refusal or withdrawal from participation.

The sample size calculation considered the total number of visits per month (*n* = 485) to CAPS during the data-collection period (Jataí: 202 [41.6%]; Mineiros: 95 [19.6%], and Rio Verde: 188 [38.8%]). Considering a significant level of 95.0% (α = 0.05), margin of error of 2%, and a standard deviation (SD) of 17.1 of caregiver overload using the ZBI score found in a previous study in the southern region of Brazil [21], the number of participants estimated was 281. The sample size was proportionally distributed among the three sites: Jataí (*n* = 117), Mineiros (*n* = 56), and Rio Verde (*n* = 108).

### Data collection

Data collection took place between June 2014 and June 2015. Participants were recruited after obtaining permission from the service coordinators and health secretaries in the respective municipalities. All eligible caregivers who were present at CAPS at the time of data collection were invited to participate in the study. The caregivers were approached at different times: when accompanying the patient to a medical appointment, when picking up a prescription and/or medication, or when participating in a family therapy group. After the objectives, methods, benefits, and potential risks of the study were explained, the caregivers who agreed to participate signed the informed consent form and, subsequently, were interviewed face-to-face.

Data collection occurred at CAPS (63.0%) or at home (37.0%), depending on the availability of the caregiver. Patients were not allowed to be present during the interviews in order to guarantee the confidentiality of the information.

### Instruments and variables

The structured questionnaire used in the study collected sociodemographic characteristics and information on risk factors of caregiver burden based on previous studies [6, 13, 21, 25–33]. The instrument was subjected to a pilot test for calibration.

In addition, the Zarit Burden Interview (ZBI) was used to evaluate caregivers’ burden [34], an instrument adapted and validated in Brazil with a sample of caregivers of older adults with psychiatric disorders, with a Cronbach’s alpha value of 0.88 [35]. It consists of 22 items that evaluate the caregiver-patient relationship, as well as the condition of the caregiver’s health, psychological well-being, finances, and social life. Responses are scored on a five-point Likert-type scale ranging from 0 to 4. For items 1–21, respondents indicate how much they endorse each statement *(0-never; 1-rarely; 2-sometimes, 3-quite frequently, and 4-nearly always*). The last item involves respondents rating how overwhelmed they feel in the role of caregiver *(0-not at all; 1-a little, 2-moderately, 3-quite a bit, 4-extremely).* The total score ranges from 0 to 88, and the higher the score, the greater the burden perceived by caregivers [35]. The present study considered as the dependent variable the total burden score measured with the ZBI*.*

The following were considered as independent variables:

(i). Patient-related characteristics: sex (male or female) as proxy of gender; age (≤ 40, 41–59 or ≥ 60 years); education (low/elementary school, middle/high school, or high/higher education); marital status (without partner or with partner); duration of patient’s illness (years); crisis in the previous month (yes or no); medical diagnostic (ICD-10).
(ii). Caregiver-relate characteristics: sex (male or female); age (≤ 40, 41–59 or ≥ 60 years); education (low/elementary school, middle/high school, or high/higher education); marital status (without partner or with partner); children (yes or no); religion (yes or no); relationship to the patient (spouse, parental, or other); self-reported chronic disease HTN and/or diabetes (yes or no); self-reported depression (yes or no); physical activity (high [≥3 times a week] vs. average [1–2 times a week] vs. low [never or almost never]); monthly family income (in dollars); living with patient (yes or no); receiving help with caregiving (yes or no); another family member with health problems needing care (yes or no); time as caregiver (years); number of days of contact with patient (per week).

### Statistical analysis

Data were analyzed using the STATA software, version 14.0. Qualitative variables were presented as absolute and relative frequencies, and quantitative as mean and standard deviation (SD) together with their respective 95% confidence intervals (95.0% CI). The normality of the quantitative variables was verified using the *Kolmogorov-Smirnov* test [36]. Cronbach’s alpha was used to verify the reliability of the ZBI, with an acceptable internal consistency above 0.7 [37].

Initially, bivariate analysis was conducted to investigate associations between the predictor variables and the study outcome. Student’s t test or analysis of variance (ANOVA), and Pearson correlation (r) were used as appropriate. Variables with *p* < 0.10 in bivariate analysis and caregiver gender as potential confounding variables were included in a multiple-linear regression model with robust variance. The tests of VIF, Breusch-Pagan/Cook-Weisberg, and Ramsey Reset were used to verify multicollinearity, homoscedasticity, and specification of model, respectively. For all tests performed, values of *p* < 0.05 were considered statistically significant.

### Ethical aspects

The study was approved by the Research Ethics Committee of the Federal University of Goiás (protocol number 22761913.4.0000.5083). Written informed consent was obtained from all participants.

## Results

A total of 295 caregivers were recruited in the study. From these, seven were not included in the study: three due to refusal, two due to withdrawal, and two due to difficulties understanding the questions. This resulted in a final sample of 281 caregivers (response rate of 97.6%): 117 from Jataí; 109 from Rio Verde; and 55 from Mineiros.

### Characteristics of patients with mental disorders

The sociodemographic and disease-related characteristics of patients assisted by caregivers are presented in Table 1. The majority of patients were female (55.2%), with low education (55.9%), and without a partner (74.7%). Of the total, only 11.7% were 60 years or older (elderly in Brazil). Regarding the diagnosis, approximately ¼ (38.8%) of patients had schizophrenia. Patient crisis and hospitalization in the previous month were reported by 24.2 and 2.1% of caregivers, respectively. The mean duration of the mental disorder was 16.59 years (SD = 13.48).

**Table 1:** Characteristics of patients with mental disorders assisted by caregivers

Variables	n	%	95.0% CI^b^
Sex			
Male	126	44.8	39.1–59.7
Female	155	55.2	49.3–60.0
Age (years)			
≤ 40	124	44.1	28.4–50.0
41–60	124	44.1	28.4–50.0
≥ 60	33	11.7	8.5–16.0
Education (years)			
Low	157	55.9	50.0–61.6
Average	107	38.1	32.6–43.9
High	17	6.0	3.8–9.5
Marital status			
Without partner	210	74.7	69.3–79.5
With partner	71	25.2	20.5–30.7
Medical diagnostic (CID-10)			
Anxiety	26	9.3	6.4–13.2
Schizophrenia	109	38.8	33.3–44.6
Bipolar Affective Disorder	56	19.9	15.7–25.0
Depression	22	7.8	5.2–11.6
Mental retardation	34	12.1	8.8–16.4
Others	34	12.1	8.8–16.4
Crisis^a^			
No	213	75.8	70.5–80.5
Yes	68	24.2	19.6–29.5
Internment^a^			
No	275	97.9	95.4–99.0
Yes	6	2.1	0.98–4.6
	Mean	SD	95.0% CI
Duration of patient’s illness (years)	16.59	13.48	15.01–18.18

^a^Previous month

^b^95.0% confidence interval

### Characteristics of caregivers

Table 2 shows the characteristics of caregivers included in the study. It was observed that the majority were women (81.1%), with low education (67.3%), with a partner (59.1%), were somewhat religious (95.2%), and had children (92.5%). As for age, 81.4% of the sample had an age equal to or greater than 40 years. The average monthly family income was US$ 615.12 (SD = 287.27).

**Table 2:** Characteristics of caregivers and caring process

Variables	n	%	95.0% CI^c^
Sex			
Male	56	19.9	15.7–25.0
Female	225	81.1	75.0–84.3
Age (years)			
≤ 40	52	18.5	14.4–23.6
41–60	119	42.3	36.7–48.2
> 60	110	39.1	33.6–45.0
Education (years)			
Low	189	67.3	61.6–72.5
Average	77	27.4	22.5–32.9
High	15	5.3	2.3–8.6
Marital status			
Without partner	115	40.9	35.6–46.8
With partner	166	59.1	53.2–64.7
Children^a^			
No	21	7.5	5.0–11.2
Yes	259	92.5	88.8–95.0
Religion^a^			
No	13	4.6	2.7–7.8
Yes	267	95.4	92.2–97.3
Relationship with the patient			
Spouse	116	41.3	35.7–47.1
Parents	41	14.6	10.9–19.2
Other	124	44.1	38.4–50.0
Chronic disease (self-report)			
No	210	74.7	69.3–79.5
Yes	71	25.3	20.5–30.7
Depression (self-report)			
No	258	91.8	88.0–94.5
Yes	23	8.2	5.5–12.0
Living with patient			
No	77	27.4	22.5–32.9
Yes	204	72.6	67.1–77.5
Physical activity			
High	78	27.8	22.8–33.3
Average	30	10.7	7.6–14.8
Low	173	61.6	55.8–67.1
Receiving help with care			
No	194	69.0	63.4–74.2
Yes	87	31.0	25.8–36.6
Another family member needing care			
No	166	59.1	53.2–64.7
Yes	115	40.9	35.3–46.8
	Mean	SD^d^	95.0% CI^c^
Family income (U$$)^b^	615.12	312.52	576.97–652.45
Time as caregiver (years)	12.13	11.44	10.79–13.48
Contact days (per week)	6.38	1.60	6.19–6.57

^a^Missing: 1

^b^Missing: 7

^c^95.0% confidence interval

^d^Standard deviation

Of the total number of caregivers, 41.3% were partners, 14.6% were parents, and 44.1% were other people. Approximately three quarters (72.6%) lived with the patient, 69.0% did not receive help from others, and 40.9% had another family member also needing care. The average time as caregiver and contact days per week was 12.13 years (SD = 11.44) and 6.38 (SD = 1.60), respectively. The proportion of caregivers with self-reported hypertension/diabetes mellitus was 25.3% and with self-reported depression was 8.2%.

### Caregiver burden

Table 3 shows the descriptive analysis of ZBI. Analysis of caregivers showed an average total score of 27.66 (SD = 14.53). The items Cronbach’s alpha was 0.874, indicating good reliability of the instrument.

**Table 3:** Descriptive analysis of Zarit Burden Interview

Zarit Burden Interview (ZBI)	Mean ± SD^a^	Median	Q1-Q3	Min-Max
1- Do you feel that your relative asks for more help than he/she needs?	1.56 ± 1.48	1.0	0.0–3.0	0.0–4.0
2- Do you feel that because of the time you spend with your relative that you don’t have enough time for yourself?	1.11 ± 1.36	0.0	0.0–2.0	0.0–4.0
3- Do you feel stressed between caring for your relative and trying to meet other responsibilities for your family or work?	1.36 ± 1.41	1.0	0.0–2.0	0.0–4.0
4- Do you feel embarrassed over your relative’s behavior?	0.54 ± 1.00	1.0	0.0–2.0	0.0–4.0
5- Do you feel angry when you are around your relative?	0.52 ± 0.92	0.0	0.0–1.0	0.0–4.0
6- Do you feel that your relative currently affects your relationship with other family members or friends in a negative way?	0.73 ± 1.17	0.0	0.0–2.0	0.0–4.0
7- Are you afraid of what the future holds for your relative?	1.74 ± 1.57	2.0	0.0–3.0	0.0–4.0
8- Do you feel your relative is dependent upon you?	2.91 ± 1.32	3.0	2.0–4.0	0.0–4.0
9- Do you feel strained when you are around your relative?	0.64 ± 1.09	0.0	0.0–1.0	0.0–4.0
10- Do you feel your health has suffered because of your involvement with your relative?	0.63 ± 1.16	0.0	0.0–1.0	0.0–4.0
11- Do you feel that you don’t have as much privacy as you would like because of your relative?	0.71 ± 1.12	0.0	0.0–2.0	0.0–4.0
12- Do you feel that your social life has suffered because you are caring for your relative?	0.58 ± 1.07	0.0	0.0–1.0	0.0–4.0
13- Do you feel uncomfortable about having friends over because of your relative?	0.59 ± 1.08	0.0	0.0–1.0	0.0–4.0
14- Do you feel that your relative seems to expect you to take care of him/her, as if you were the only one he/she could depend on?	2.44 ± 1.61	3.0	1.0–4.0	0.0–4.0
15- Do you feel that you don’t have enough money to care for your relative, in addition to the rest of your expenses?	2.30 ± 1.53	2.0	1.0–4.0	0.0–4.0
16- Do you feel that you will be unable to take care of your relative much longer?	1.17 ± 1.42	0.0	0.0–2.0	0.0–4.0
17- Do you feel you have lost control of your life since your relative’s illness?	0.79 ± 1.24	0.0	0.0–2.0	0.0–4.0
18- Do you wish you could just leave the care of your relative to someone else?	0.57 ± 1.09	0.0	0.0–1.0	0.0–4.0
19- Do you feel uncertain about what to do about your relative?	1.33 ± 1.39	0.0	1.0–2.0	0.0–4.0
20- Do you feel you should be doing more for your relative?	2.40 ± 1.44	2.0	2.0–4.0	0.0–4.0
21- Do you feel you could do a better job in caring for your relative?	2.33 ± 1.44	2.0	1.0–4.0	0.0–4.0
22- Overall, how burdened do you feel in caring for your relative?	1.25 ± 1.22	1.0	0.0–4.0	0.0–4.0

^a^Standard deviation

### Factors associated with caregiver burden

#### Bivariate analysis

Table 4 presents the results of the bivariate analysis of the potential factors related to the characteristics of patients with caregiver burden. It was observed that ZBI scores were significantly higher in male patients when compared to female patients (*p* < 0.001). Additionally, scores were higher in patients with recent crisis when compared to patients without this characteristic (*p* < 0.001).

**Table 4:** Bivariate analysis of the potential factors related to the characteristics of patients with caregiver burden

Variables	ZBI Score	*p*
	Mean ± SD^a^	
Sex		
Male	29.76 ± 13.97	0.028
Female	25.95 ± 14.79	
Age (years)		
≤ 40	28.37 ± 14.74	0.320
41–60	27.89 ± 14.60	
≥ 60	24.12 ± 13.32	
Education (years)		
Low	29.20 ± 14.81	0.117
Average	25.99 ± 13.31	
High	23.94 ± 18.03	
Marital status		
Without partner	28.35 ± 14.14	0.169
With partner	25.61 ± 15.54	
Medical diagnostic (ICD-10)		
Anxiety	26.65 ± 15.67	0.153
Schizophrenia	29.19 ± 14.17	
Bipolar Affective Disorder	25.07 ± 14.15	
Depression	24.82 ± 12.28	
Mental retardation	25.21 ± 13.13	
Others	32.06 ± 17.13	
Crisis		
No	25.84 ± 14.19	< 0.001
Yes	33.37 ± 14.17	
Internment		
No	27.47 ± 14.36	0.147
Yes	36.17 ± 20.66	
	r^b^	*p*
Duration of patient’s illness (years)	0.045	0.448

^a^Standard deviation

^b^Pearson’s correlation coefficient

Table 5 presents the results of the bivariate analysis of potential factors characteristic of caregivers and the caring process associated with the outcome. The factors showing statistically significant relationships with caregiver burden in this analysis included age (*p* = 0.010), relationship to the patient (*p* = 0.016), self-reported depression (*p* < 0.001), receiving help with caregiving (*p* = 0.002), another family member needing care (*p* = 0.033), and living with the patient (*p* = 0.045). Furthermore, time as caregiver (*p* = 0.039) and number of contact days per week (*p* < 0.001) were correlated positively with the overload scores.

**Table 5:** Bivariate analysis of potential factors characteristic of caregivers and the caring process associated with caregiver burden

Variables	ZBI Score	*p*
	Mean ± SD^a^	
Sex		
Male	26.18 ± 15.67	0.395
Female	28.03 ± 14.24	
Age (years)		
≤ 40	22.33 ± 12.34	0.010
41–60	28.22 ± 14.95	
> 60	29.57 ± 14.53	
Education (years)		
Low	28.49 ± 13.71	0.136
Average	24.97 ± 16.35	
High	30.81 ± 13.63	
Marital status		
Without partner	27.55 ± 14.42	0.916
With partner	27.73 ± 14.64	
Children		
No	25.19 ± 19.35	0.439
Yes	27.73 ± 13.96	
Religion		
No	27.37 ± 16.49	0.016
Yes	30.48 ± 13.23	
Relationship with the patient	25.11 ± 14.63	
Spouse		
Parents	27.53 ± 14.85	0.805
Other	28.03 ± 13.63	
Chronic disease (self-report)		
No	26.13 ± 13.52	< 0.001
Yes	44.78 ± 14.71	
Depression (self-report)		
No	21.69 ± 13.30	0.123
Yes	28.04 ± 14.50	
Living with patient		
No	24.67 ± 15.78	0.096
Yes	29.57 ± 10.50	
Physical activity	28.68 ± 14.41	
High		
Average	23.67 ± 13.80	0.002
Low	29.45 ± 14.52	
Receiving help with care		
No	26.12 ± 14.09	0.033
Yes	29.88 ± 14.92	
Another family member needing care		
No	24.83 ± 13.84	0.045
Yes	28.73 ± 14.67	
	r^b^	*p*
Family income (U$$)	−0.073	0.229
Time as caregiver (years)	0.123	0.039
Contact days (per week)	0.219	< 0.001

^a^Standard deviation

^b^Pearson’s correlation coefficient

#### Multivariable analysis

All variables with *p* value < 0.10 in the bivariate analysis and caregiver gender were included in a multiple-regression model for control of potential confounders. In the multivariable analysis, the following factors were independently associated with caregiver burden: age over 60 years (β = 4.55, *p* = 0.044); self-reported depression (β = 15.14, *p* < 0.001); not receiving help with caregiving (β = 4.69, *p* = 0.004); having another family member needing care (β = 3.28, *p* = 0.047); patient crisis in the last 30 days (β = 6.80, *p* < 0.001); and days of contact per week (β = 1.70, *p* = 0.001) (Table 6). The model explained 23.3% of the variation of ZBI scores (adjusted R^2^: 0.233).

**Table 6:** Factors associated with caregiver burden

Variables	β^a^	95% CI^b^	*p*
Age (years)			
≤ 40	Ref.^c^		
41–60	3.33	−0.81; 7.47	0.115
> 60	4.55	0.11; 8.99	0.044
Depression (self-report)			
No	Ref.^c^		
Yes	15.14	8.52; 21.75	< 0.001
Receiving help with care			
Yes	Ref.^c^		
No	4.69	1.55; 7.83	0.004
Another family member needing care			
No	Ref.^c^		
Yes	3.28	0.04; 6.33	0.047
Patient crisis			
No	Ref.^c^		
Yes	6.80	3.24; 10.35	< 0.001
Contact days per week	1.70	0.68; 2.73	0.001

^a^Regression coefficient

^b^95.0% Confidence interval

^c^Reference category

*R* ^2^: 0.274

Adjusted *R* ^2^: 0.233

VIF Mean: 1.48

Breusch-Pagan, Cook-Weisberg test: *x* ^2^: 1.02; *p* = 0.311

Skewness *x* ^2^: 21.99; *p* = 0.108

Kurtosis: *x* ^2^: 1.29; p = 0.256

Ramsey test: F (2262) = 0.64; *p* = 0.589

## Discussion

The present study analyzed the factors associated with burden in caregivers of patients with mental disorders treated in community services, using data from a sample from three municipalities in Goiás state (Central-West Region of Brazil), thereby contributing to the knowledge about caregiving burden in this population in country. The results show caregiver burden of mild to moderate in sample [38]. Additionally, it was found that self-reported depression, not receiving help with care, more days of contact per week with patient, having another member of the family who needs care, and recent crisis by the patient were factors associated with caregiver burden in sample.

In this study, the socio-demographic characteristics of the caregivers’ sample are similar to those in other studies with caregivers of patients with mental disorders at national [21, 33, 39, 40], which verified the predominance of female caregivers, with low education and over 40 years old.

The analysis of caregiver burden showed a mean score of 27.66 points on the ZBI. The results of previous studies have shown wide variation in caregiver burden worldwide measured by ZBI, which can be attributed to the methodological differences between the studies, cultural or behavioral factors between countries, and socio-demographic characteristics of caregivers. For example, Shamsaei et al. [8] found an average score of 51.73 points in psychiatric patients in Iran. In Turkey, scores of 68.64 were found among caregivers [41]. In Nigeria, studies have shown a variation from 40.98 [42] to 41.32 [43] points on burden of caregivers of patients with mental disorders. A study conducted in the southern region of Brazil found an average score of 44.2 points in caregivers of 406 patients with mental disorders [21].

In this study, caregiver burden was associated with being over 60 years of age. The gradual advancement of age decreases physiological reserves, increases the risk of disease, and leads to a decline in individuals’ intrinsic capacities. However, the effects of age are mostly derived from the physical and social environments that influence life, particularly when the circumstances have a cumulative impact on health [44]. Caring for patients with mental disorders can become a source of stress and generate burden in older caregivers [45]. Other studies also show association between higher age and caregiver burden in caregivers of patients with mental disorders [5, 14, 28].

In the present study, not receiving help with caregiving was shown to be associated with burden. In a study in São Paulo, located in the southeast region of Brazil, informal social support was an important aspect to minimize the burden of caring for dependents [32], and lack of other family members helping caregivers to care for individuals with mental disorders generated higher burden in studies conducted in Istanbul, Turkey [46]; Stockholm, Sweden [47]; and Bangalore, India [28]. Furthermore, higher burden levels were found in caregivers of patients with mental disorders who perceived less family support in Singapore [48] and the United States [6]. The role of caring for a family member with a mental disorder usually falls on one person and can have negative consequences on their physical, psychological, and social health. Situations where the primary caregiver does not have the support of other family members to share the care can lead to increased burden.

There was also an association between caregiver burden and having another family member with health problems requiring care. In fact, caregivers who have more than one dependent with health problems show higher levels of burden [21]. It is assumed that this may occur because of the increase in caregiving activities for sick family members, which may enhance the negative effects of caregiving.

Our investigation found an association between the number of days of contact with the patient and caregiver burden. In fact, family members living with the patient or spending long periods of time with him or her were found to experience more caregiver burden [11, 13, 30, 31]. Burden was shown to increase with higher frequency and intensity of contact with patients with bipolar disorder or when patients lived with caregivers [13]. It has been shown that the longer the contact time with the patient, the higher the anxiety levels the caregiver experienced [48]. The caregiver’s contact with the patient requires attention, availability, patience, and resistance to care for him or her. However, if these qualities are not present in the care provided, this can produce negative feelings and stress, increasing caregiver burden.

The present study investigated whether the patient had suffered a crisis anytime within the 30 days prior to conducting the study and examined how this affected caregivers’ burden. Mental health crises are characterized by disorganization or violent behavior, as well as expressions of isolation, sadness, apathy, and insecurity. These behaviors can produce feelings of uncertainty, threat, and fear in both patients and those who live with them. The resulting severe difficulties in communication and expression between those involved can amplify the problem and generate a sense of urgency. It is difficult for families to live with a member in intense psychological crisis, due to the disorganization of internal and external relations, and this may give rise to a mixture of feelings, often contradictory and sometimes even unbearable [49]. Patients with mental disorders exhibiting symptoms and crisis requiring a higher level of institutionalization were associated with greater caregiver burden [46]. The symptoms of mental disorders as well as patients’ behavior may change during the course of the disease, restricting their functional capacity and at times, generating situations that require more attention and assistance, which can increase burden. However, when patients’ functional capacity permits greater independence in activities of daily living, this can facilitate the effectiveness of the caregivers’ job. This was verified in a study in Sweden, which found that the greater the patient’s ability to function, the less burden and better health status of the caregiver, as the impact of the patient’s condition has a direct effect on the stress experienced by the caregiver [29, 47].

The present study found an association between self-reported depression and caregiver burden. In Salvador, located in the northeast macro-region of Brazil, caregivers of children with mental disorders had a depression prevalence of 47.5%, with a predominance of anxiety disorders, followed by mood disorders, and substance abuse [50]. In Sao Paulo, a population-based survey found that 44.8% of individuals experienced at least one mental disorder during their lives, and depression was one of the most prevalent [51]. Additionally, studies conducted in other countries confirm the findings of this investigation. A study in Ontario, Canada on mental health in informal caregivers found that they showed a significantly higher prevalence of mental disorders than did the general population [52]. In a multicenter study in the United States, those caregivers of patients with bipolar disorder, who experienced higher levels of burden, were more likely to experience depression [6]. In a multicenter study conducted in five European countries (France, Germany, Italy, Spain, and the United Kingdom), caregivers of patients with schizophrenia reported a greater severity of depressive symptoms and were more likely to use prescription drugs for depression than were non-caregivers and caregivers of patients with other diseases [53]. In a study conducted in India, 1/3 of caregivers of patients with schizophrenia had psychological morbidity and reported more levels of burden [30]. In Konya, Turkey, family caregivers of patients with obsessive-compulsive disorder had an average burden score greater than that of controls, and in regression analysis, depression was an independent predictor of burden, suggesting that depression may cause a disruption in social and occupational functioning, and increase burden [54]. Moreover, a recent longitudinal survey of caregivers of patients with bipolar disorder conducted in the United States concluded that burden predicts depression, being an important indicator over time and supporting the hypothesis of this study. This suggests that caregivers who experience high levels of burden are probably those already suffering from depression due to increased family burden and/or other life events (e.g., trauma or loss), in addition to genetic vulnerability [6]. Depression has deleterious effects on quality of life, affecting physical, mental, social, and environmental health, as well as increasing the risk of disease [25]. Thus, depression may be a two-way street: on the one hand, burden can increase depression symptoms; on the other hand, depression may enhance burden. Therefore, basic care should involve interventions, such as family therapy groups, psychoeducational programs, and other strategies in order to prevent burden and minimize the risk of development or recurrence of depression in caregivers of patients with mental disorders.

The interpretation of our results should be considered with the following limitations. The cross-sectional nature of the investigation did not allow the establishment of causal relations between the outcome and the variables investigated. Longitudinal studies are more suitable to verify the predictors of caregiver burden. The representativeness of the sample can be questioned and participants not being randomly selected may have produced unperceived biases. The data were self-reported, thus being liable for response and memory bias. Furthermore, the prevalence of depression, where not confirmed by physicians, may have been underestimated among caregivers. The participants were caregivers of patients that attended public health services located in a specific region, which makes it difficult to generalize the results. Although the diagnosis of patients was collected from their medical records, these data are subject to information or interpretation bias. The difficulty of identifying other studies using a similar methodological design and the same instrument to measure burden compromised the comparison of the current results with those of Brazilian studies. Despite the above limitations, this study identified factors that are closely related to caregiver burden, and can contribute to strategies and interventions to reduce the burden experienced by caregivers of patients with mental disorders.

## Conclusions

This study identified factors associated with burden in caregivers of patients with mental disorders. The association of burden with the following factors were found: self-reported depression, more than 60 years of age, not receiving help with caregiving, having another family member with health problems needing care, and more days of contact with the patient per week. These findings may lead to discussions and actions, which, in the short or medium term, may help to reduce the effects of burden on the caregivers of patients with mental disorders.

More studies are necessary to better understand the phenomenon of caregiver burden in Brazil. Longitudinal cohort studies should be performed to deepen our understanding of the variables involved in burden and its effects on caregivers. In addition, the psychosocial network of the public mental health services should offer strategies and interventions, such as family therapy groups, psycho-education programs, educational lectures, and physical and leisure activities for caregivers to help prevent or minimize the effects of burden in this population. Finally, family caregivers should be taken into account by professionals in the psychosocial network. After all, they are an important part of the mental-health field, providing home care to patients with mental disorders.

## Abbreviations

CAPS: Brazilian Psychosocial Care Centers (*Centros de Atenção Psicossocial*)
HTN: Arterial hypertension
ICD-10: International Classification of Diseases
ZBI: Zarit Burden Interview
